# Supplementary figures and images for: Pharmacokinetics of hydrogen administered intraperitoneally as hydrogen-rich saline and its effect on ischemic neuronal cell death in the brain in gerbils
Source: PLoS One. 2022 Dec 27;17(12):e0279410. doi: 10.1371/journal.pone.0279410 (PMC9794077; doi:10.1371/journal.pone.0279410)

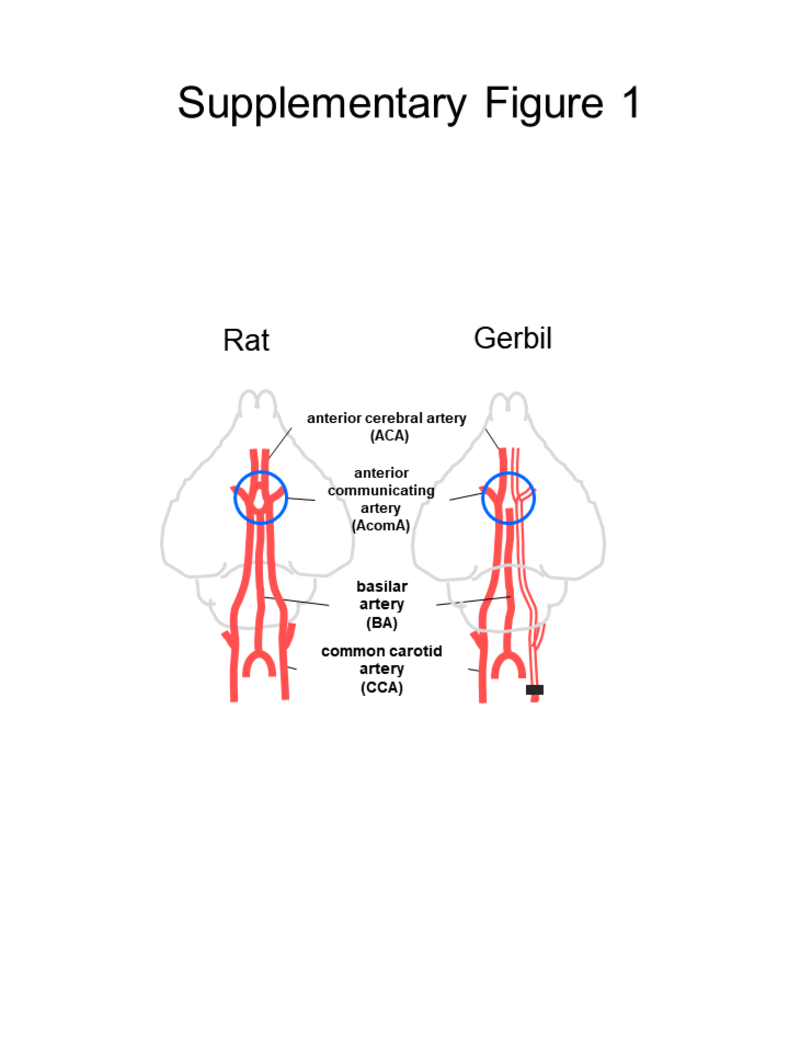

Supplement: S1 Fig — In rats (left), the internal carotid artery system, vertebral artery system, and left and right medial artery systems are connected at the base of the brain to form the circle of Willis. Therefore, if one artery is occluded, blood can still flow to the brain via the other vessels. In the gerbil (right), the basilar arterial ring lacks anastomoses between the right and left internal carotid arteries and between the carotid and vertebral arteries, so occlusion of the common carotid artery can easily induce ipsilateral cerebral ischemia. (TIF) [file pone.0279410.s001.tif]

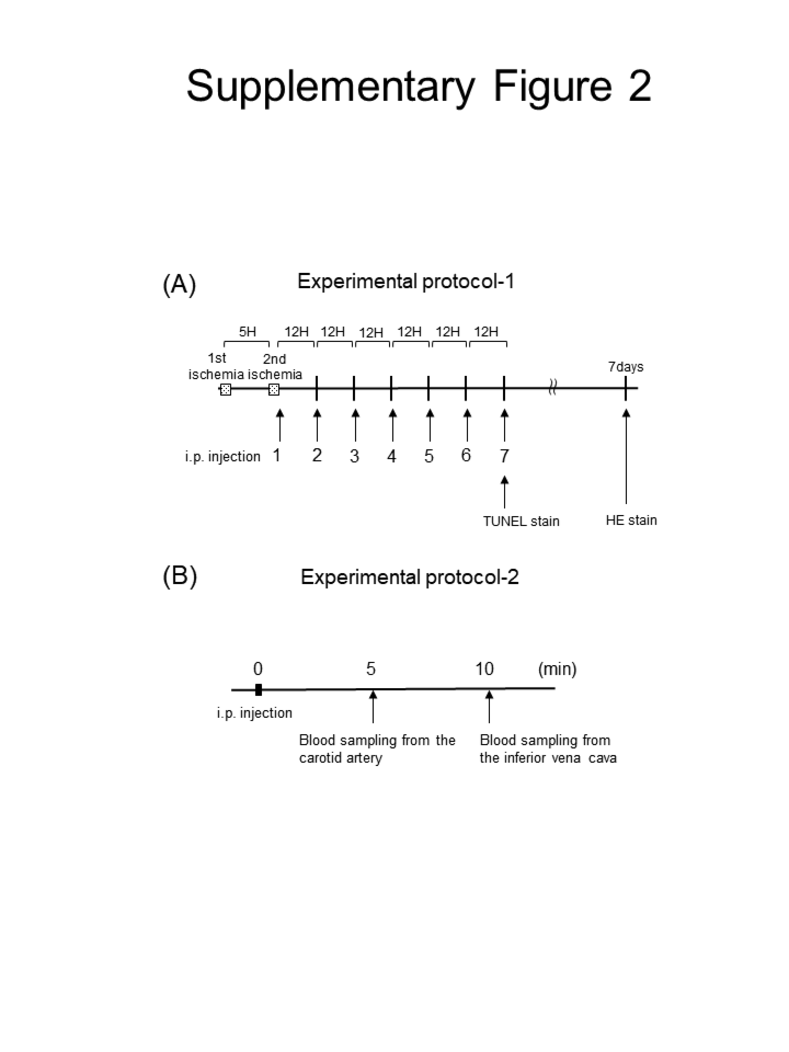

Supplement: S2 Fig — (A) Experimental protocol-1. After two 10-minute occlusions of the left carotid artery, the animals were randomly divided into H2-rich saline and saline groups. Animals in each group received 3 or 7 intraperitoneal injections of 20 ml/kg body weight of each solution. Histological analysis was performed on days 3 and 7. (B) Experimental protocol-2. H2-rich saline or saline solution (20 ml/kg BW) was administered intraperitoneally once. Blood was drawn from the carotid artery after 5 minutes and from the IVC after 10 minutes, and H2 concentration was measured by gas chromatography. (TIF) [file pone.0279410.s002.tif]

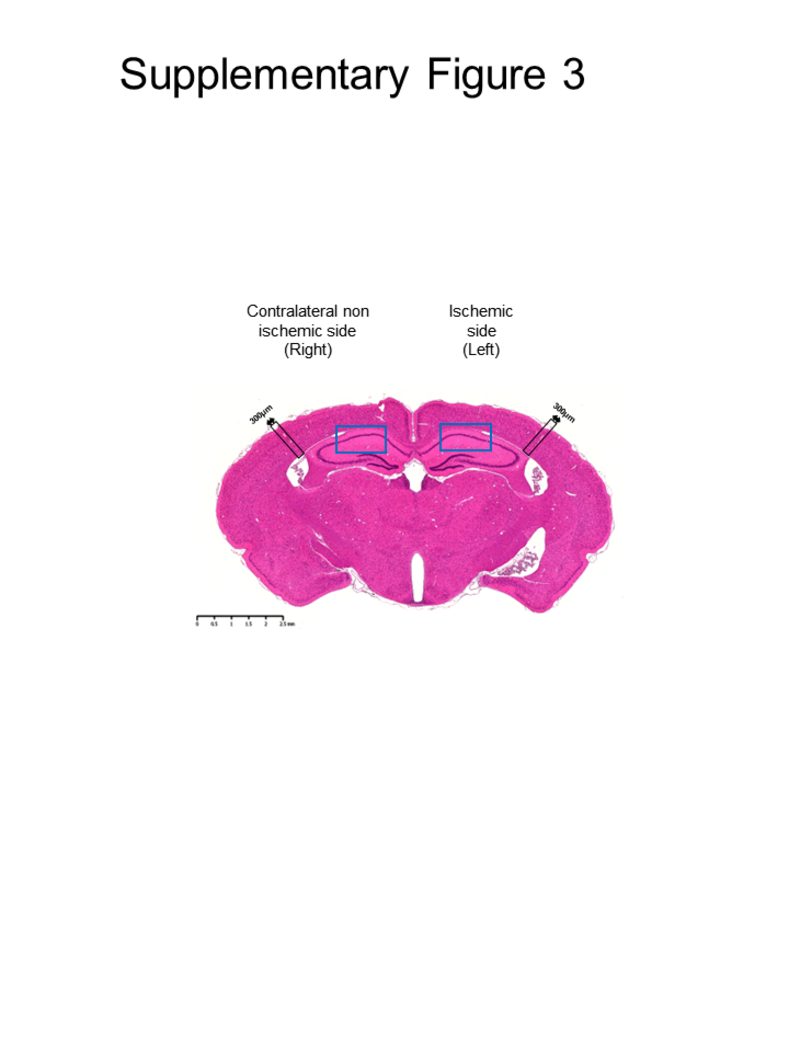

Supplement: S3 Fig — Cerebral histology of the cerebrum of a gerbil coronally sectioned at the levels of the infundibulum and stained with hematoxylin and eosin. Neuronal cells were counted in the hippocampal CA1 region (areas inside the blue rectangles) and the central part of the cerebral cortex (areas inside the black rectangles, 300 μm wide) in the left and right cerebral hemispheres. (TIF) [file pone.0279410.s003.tif]

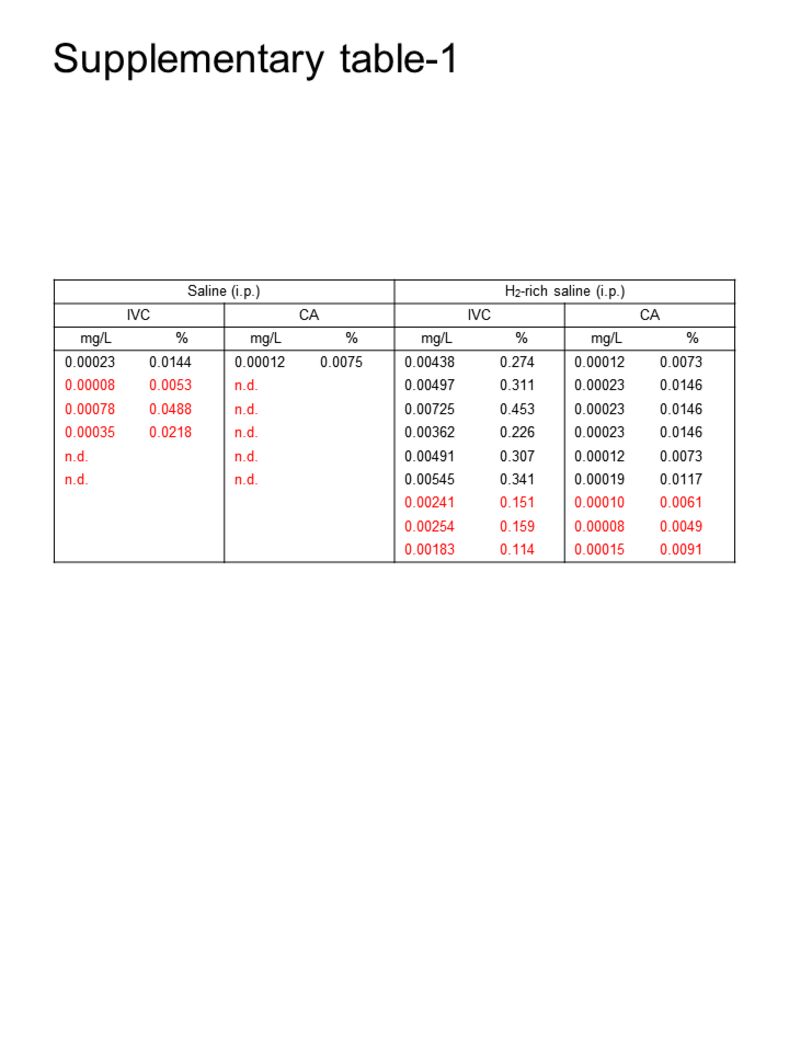

Supplement: S1 Table — H2 measurements are shown in concentration (mg/L) and saturation (%). Because H2 dissolves at 1.6 mg/L under 0.1 MPa, the H2 saturation of each sample was converted by assuming 1.6 mg/L as 100% saturation. IVC, inferior vena cava, CA carotid artery, n.d., no detected, i.p., intraperitoneal injection. (TIF) [file pone.0279410.s004.tif]
