# Supplementary material for: Pharmacokinetics of hydrogen administered intraperitoneally as hydrogen-rich saline and its effect on ischemic neuronal cell death in the brain in gerbils
Source: PLoS One. 2022 Dec 27;17(12):e0279410. doi: 10.1371/journal.pone.0279410 (PMC9794077; doi:10.1371/journal.pone.0279410)
Supplement: Attachment — Submitted filename: Response letter_PLOSONE_edited_20221104.docx [file pone.0279410.s005.docx]

Alexander A. Mongin, Ph.D.
Academic Editor
PLOS ONE

November 7, 2022

Dear Dr. Mongin:

On behalf of my co-authors, I am pleased to resubmit our manuscript “**Hydrogen indirectly inhibits ischemic neuronal cell death in the gerbil brain after intraperitoneal administration of hydrogen-rich saline**” (Submission ID PONE-D-22-22387) to be reconsidered for publication in PLOS ONE. In response to suggestions from the academic editor and reviewers, we have changed the title to " **Pharmacokinetics of hydrogen administered intraperitoneally as hydrogen-rich saline and its effect on ischemic neuronal cell death in the brain in gerbils.”**

First, we would like to express our sincere gratitude for the effort you put into deciding on the academic editor and reviewers for this paper. They appeared to be very knowledgeable about the topic of our manuscript. We acknowledge the concerns raised by the academic editor and reviewers and thank them for their time and effort. Their comments have guided us in restructuring the manuscript, and we believe that the text has been greatly improved by their constructive feedback.

We address each of the academic editor’s and reviewers’ comments in a point-by-point manner below.

We hope that you consider our revised manuscript suitable for publication in PLOS ONE, but please let us know if you have any questions or concerns.

Yours sincerely,

Motoaki Sano, MD, PhD

On behalf of all authors

Chairman, Center for Hydrogen Medicine,

Vice Chair and Associate Professor, The Department of Cardiology,

Keio University School of Medicine

35 Shinanomachi, Shinjuku-ku, Tokyo 160-8582, Japan

Tel: +81-(3)-5363-3874, Fax: +81-(3)-5363-3875

E-mail: msano@a8.keio.jp

**Point-by-point response to the academic editor’s and reviewers’ comments**

Thank you for your constructive comments and suggestions, which have helped to improve our manuscript. Our point-by-point responses are shown below:

**Responses to the Academic Editor and Reviewers**

*#1 Academic Editor*

*Your publication does not adhere to PLOS ONE Data Sharing Policy, as your primary results have not been included in supplemental files or made available through open database depository*

**Author Response:** In accordance with the PLOS ONE Data Sharing Policy, we have revised all bar graphs as bar graph + error bars + dot blots to clearly show individual data (Figure 1 to Figure 4). The results of the hydrogen concentration measurements are shown in Figure 5 as bar graph + error bars + dot blots and were statistically analyzed. These raw data are presented in the supplementary table.

*#2 Reviewer 2 comment*

*Your baseline hydrogen measurement was done only once.  This is insufficient for making conclusions and statistical comparisons.   Please add additional baseline measurements and run statistics comparisons.*

**Author Response:** To clarify the changes in blood hydrogen concentration after intraperitoneal administration of H_2_-rich saline, 3 new animals were added to the hydrogen group (total: 9 animals) and 5 animals to the saline group (total: 6 animals). The n.d. (not detectable) point was calculated as zero for statistical tests. The raw data of the hydrogen concentration measurements are shown in the supplementary table, and to clarify the experimental results, we created a new Figure 5 showing the results of the statistical tests.

*#2 Reviewer 1 comment*

*Sensitivity of the method may not be sufficient to detect increases in the intracarotid hydrogen levels. Please add additional discussion in the text.*

**Author Response:** To measure blood H_2_ concentration, we first inserted a needle into the rubber lid of a 13.5-mL sealed vial, extracted 1 mL of air, and injected 1 mL of blood. To prevent outgassing, we immediately applied wax to the rubber lid to seal the injection hole. In such a sealed vial, H_2_ in the blood is released into the air phase because the air in the vial contains almost no H_2_ gas, so after injection of blood, most of the H_2_ gas moves from the blood (liquid phase) into the air (gas phase). Therefore, examining the concentration of H_2_ gas in the air inside the vial enabled the H_2_ concentration in the blood to be estimated. Some of the air phase (0.2 mL, 0.4 mL, or 1 mL, depending on the H_2_ concentration) was collected from the vial, and the H_2_ concentration was measured by gas chromatography (TRIlyzer mBA-3000, Taiyo, Co., Ltd.). A calibration curve was obtained by using H_2_ gas at a concentration of 0 (nitrogen gas), 5, 50, and 130 parts per million (ppm). The detectable value of the analyzer is 0.1 ppm (i.e., the analyzer shows values of 0.1 ppm or higher), and the limit of quantitation is considered to be approximately 0.2 ppm.

Samples were collected from the carotid artery after 5 minutes and from the inferior vena cava after 10 minutes, and each sample was measured twice. At the same time, air was collected from the exact same location and injected into a blood-free vial, and the H_2_ gas concentration was measured. The value of H_2_ in the air was 0.5 ppm, i.e., above the limit of quantitation. The air value was subtracted from each measured sample value to obtain a corrected value. Thus, only samples with an H_2_ value above 0.5 ppm contained H_2_ also from blood.

In the revised manuscript, the Methods section describes in detail the method used for measuring hydrogen concentration. In the Discussion section, we also explain the sensitivity of the H_2_ concentration measurement.

*#2 Academic Editor comment*

*Due to short half-life of hydrogen in arterial circulation (~90 seconds), it is not clear if the selected timing for the intraarterial hydrogen measurements truly reflect transients of this gas upon intraperitoneal delivery in gerbils. Please additionally discuss this in the text.*

**Author Response:** Thank you for pointing out this important point. In a previous study, when we administered an H_2_-rich solution to the jejunum of minipigs, we observed that the H_2_ concentration in the portal blood was increased at 2 minutes and continued to rise to 2 minutes and then remained at approximately the same level at 5, 10, 20, and 30 minutes. Therefore, it is unlikely that the timing of the peak carotid H_2_ concentration was missed. In the revised manuscript, we now explain the above in Discussion section to justify the timing of the H_2_ concentration measurement.

*#3 Academic Editor comment*

*In Fig. 1 and Fig 2, please include additional insets with high magnification allowing for better discrimination between healthy and dying cells and include markings indicating healthy and injured/dead cells.*

**Author Response:** Thank you for this suggestion. We have added a high magnification image in Figure 1-4 and have marked healthy and dying cells in it so that the reader can distinguish between them.

*#4 Reviewer 1 comment*

*TUNEL staining quantification was performed in context but not vulnerable hippocampus. If the relevant IHC slides/scans are preserved, please add hippocampal quantification or explain why it is impossible.*

**Author Response:** We have followed the Reviewer's instructions and added the results of TUNEL staining of the hippocampus as Figure 3. The number of TUNEL-positive cells in the hippocampus was measured and compared between the two groups. At day 3 of ischemia-reperfusion injury, there was no significant decrease in the number of TUNEL-positive cells after intraperitoneal administration of H_2_-rich saline.

*#5 Reviewer 1, Review 2, Academic Editor comments*

*Please exclude references to indirect mechanisms and gut-brain interactions from abstract, discussion, and concluding statements in the manuscript.*

**Author Response:** As directed by Reviewer 1, Reviewer 2, and the Academic Editor, we have excluded statements on indirect mechanisms and gut-brain interactions, as well as the cited references. We have completely rewritten the Discussion.

#5 Reviewer 1 comment

*If an indirect effect is to be judged, research evidence of such an effect should be provided, rather than simply relying on insufficient and rigorous evidence to guess.*

**Author Response:** We completely agree with this comment. As directed by Reviewer 1, Reviewer 2, and the Academic Editor, we have excluded statements on indirect mechanisms and gut-brain interactions, as well as the cited references. Intraperitoneal administration has been the route of administration most frequently used for observing the effects on rodents of H_2_ in H_2_-rich saline. To the best of our knowledge, no studies have evaluated the pharmacokinetics of H_2_ administered by this route. We hope that our study will help to elucidate the mechanism of action of H_2_.

*HE staining, but* TUNEL *staining only in the cortex, there are no staining in hippocampus, which is not readily understood.*

**Author Response:** We have added **t**he results of TUNEL staining of the hippocampus as Figure 3. The number of TUNEL-positive cells in the hippocampus was measured and compared between the two groups. At day 3 of ischemia-reperfusion injury, there was no significant decrease in the number of TUNEL-positive cells after intraperitoneal administration of H_2_-rich saline.

#5 Reviewer 2 comment

*The study is confirmative. The reported data just confirm previously published data but on a different rodent stroke model and no new scientific information is reported. The manuscript would benefit from addressing the mechanism for H2 protective effect.*

**Author Response:** Intraperitoneal administration has been widely used as a route of administration for observing the brain-protective effects in rodents of H_2_ in H_2_-rich saline. To our knowledge, this article is the first to report on the pharmacokinetics of H_2_ administered by this route of administration. In addition, this study is novel in that it examined the effects of intraperitoneal administration of H_2_-rich saline on individual neuronal death in the hippocampus and cerebral cortex—rather than reduction in the size of necrotic brain tissue—in a gerbil experimental system, which is a reliable model of cerebral ischemia.

*Table 1 data (H2 concentration): n=1 for saline group is not sufficient; statistical analysis is not performed.*

**Author Response:** We completely agree with this comment. To clarify the changes in blood hydrogen concentration after intraperitoneal administration of H_2_-rich saline, we added 3 new animals to the H_2_ group (total: 9 animals) and 5 to the saline group (total: 6 animals). The n.d. (not detectable) point was calculated as zero for statistical tests. The raw data of the hydrogen concentration measurements are shown in the supplementary table, and to clarify the experimental results, we created a new Figure 5 showing the results of the statistical tests.

*Minor suggestions:*

*Blood H_2_ concentration assay should be described in more details.*

**Author Response:** Thank you for your important comment. To measure blood H_2_ concentration, we first inserted a needle into the rubber lid of a 13.5-mL sealed vial, extracted 1 mL of air, and injected 1 mL of blood. To prevent outgassing, we immediately applied wax to the rubber lid to seal the injection hole. In such a sealed vial, H_2_ in the blood is released into the air phase because the air in the vial contains almost no H_2_ gas, so after injection of blood, most of the H_2_ gas moves from the blood (liquid phase) into the air (gas phase). Therefore, examining the concentration of H_2_ gas in the air inside the vial enabled the H_2_ concentration in the blood to be estimated. Some of the air phase (0.2 mL, 0.4 mL, or 1 mL, depending on the H_2_ concentration) was collected from the vial, and the H_2_ concentration was measured by gas chromatography (TRIlyzer mBA-3000, Taiyo, Co., Ltd.). A calibration curve was obtained by using H_2_ gas at a concentration of 0 (nitrogen gas), 5, 50, and 130 parts per million (ppm). The detectable value of the analyzer is 0.1 ppm (i.e., the analyzer shows values of 0.1 ppm or higher), and the limit of quantitation is considered to be approximately 0.2 ppm.

Samples were collected from the carotid artery after 5 minutes and from the inferior vena cava after 10 minutes, and each sample was measured twice. At the same time, air was collected from the exact same location and injected into a blood-free vial, and the H_2_ gas concentration was measured. The value of H_2_ in the air was 0.5 ppm, i.e., above the limit of quantitation. The air value was subtracted from each measured value to obtain a corrected value. Thus, only samples with a value above 0.5 ppm contained H_2_ also from blood.

In the revised manuscript, the Methods section describes in detail the method of measuring hydrogen concentration. In the Discussion section, we also explain the sensitivity of the H_2_ concentration measurement.
